# Supplementary material for: Identification of four unconventional kinetoplastid kinetochore proteins KKT22–25 in Trypanosoma brucei
Source: Open Biol. 2019 Dec 4;9(12):190236. doi: 10.1098/rsob.190236 (PMC6936259; doi:10.1098/rsob.190236)
Supplement: Supplemental Material [file rsob190236supp1.pdf]

Supplemental Materials for

Identification of four unconventional kinetoplastid kinetochore proteins KKT22–25

in *Trypanosoma brucei*

Olga O. Nerusheva, Patryk Ludzia, and Bungo Akiyoshi

Supplemental Figures

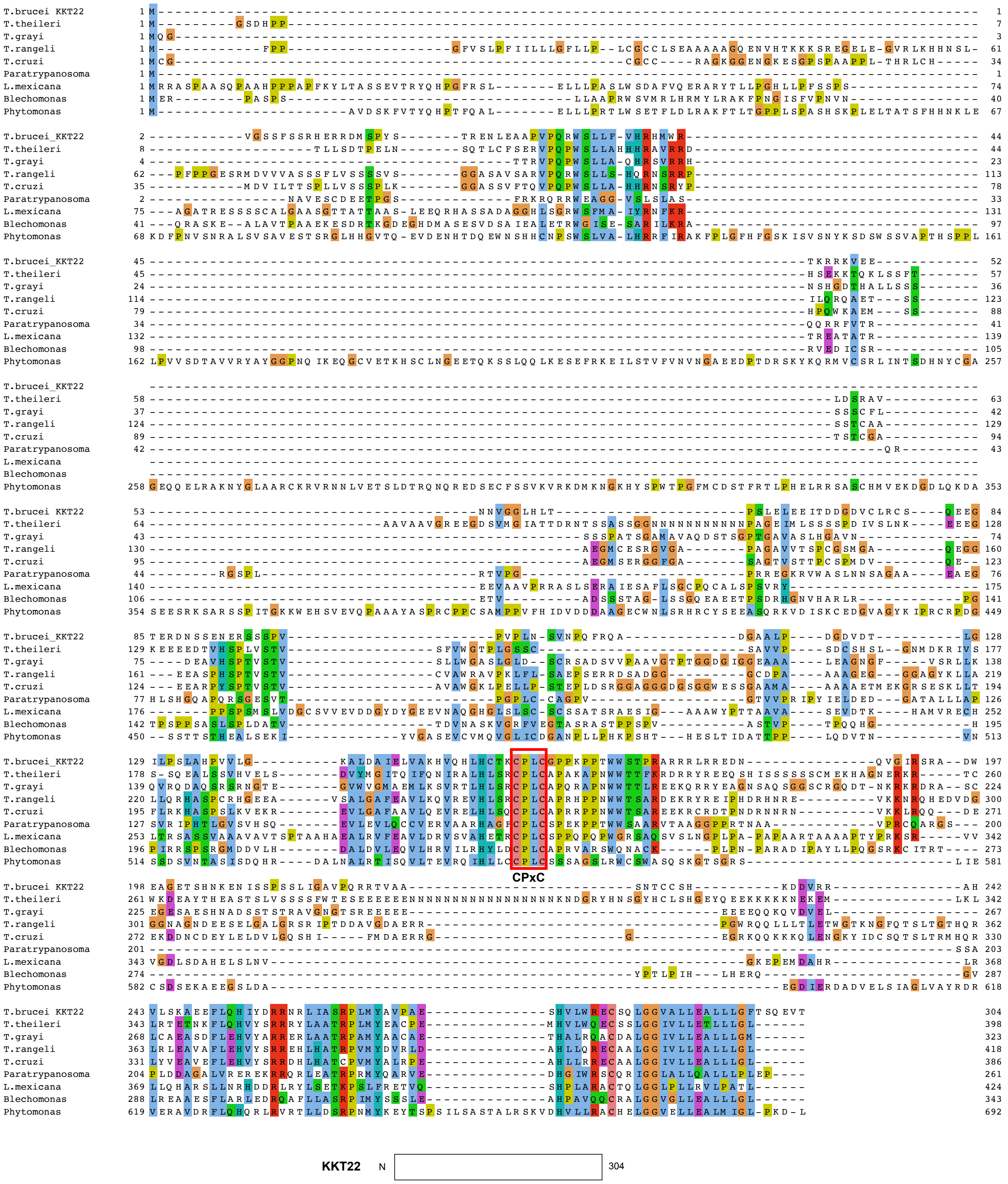

Figure S1. Multiple sequence alignment of KKT22

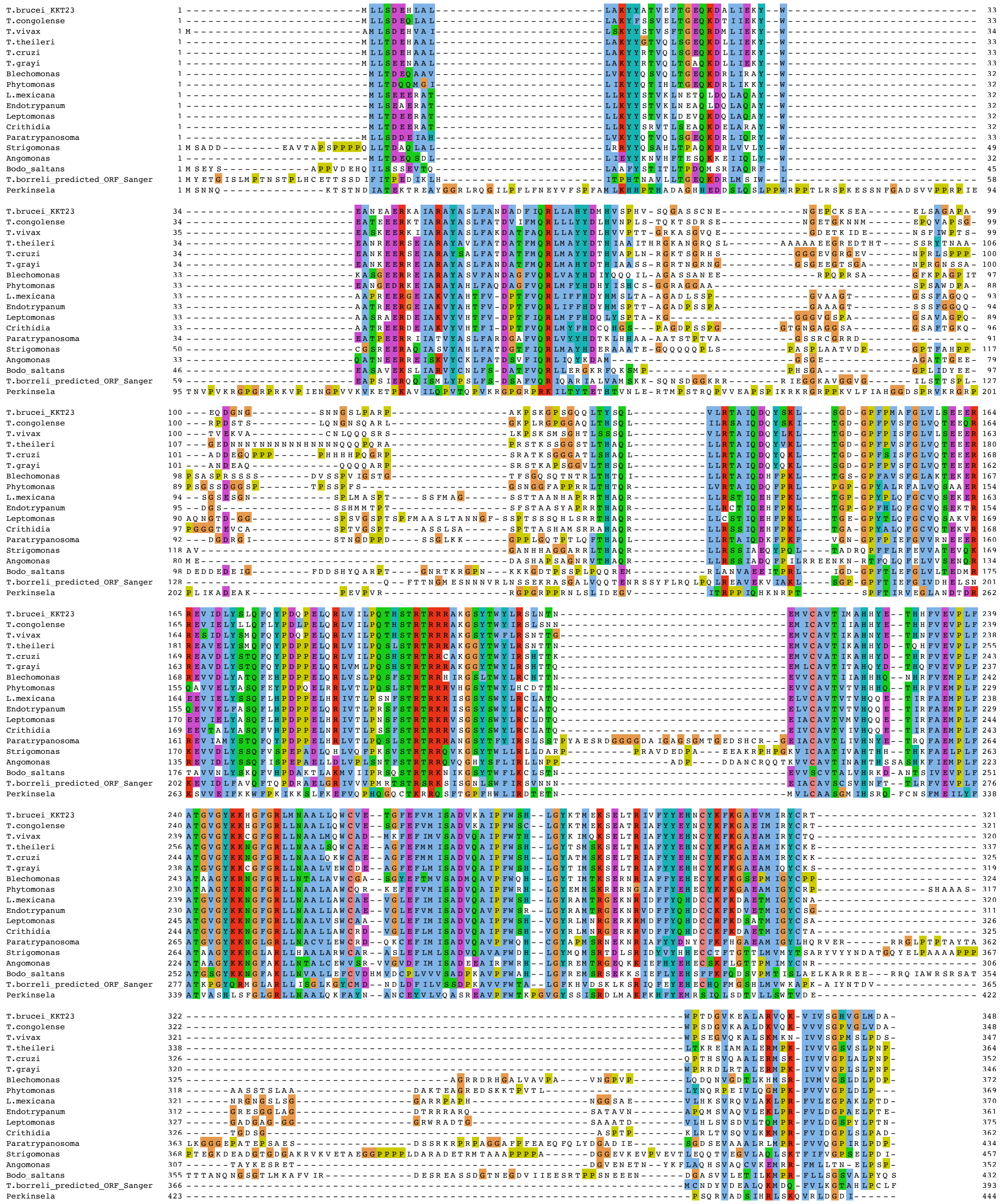

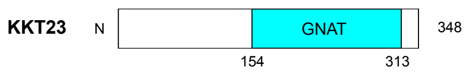

Figure S2. Multiple sequence alignment of KKT23

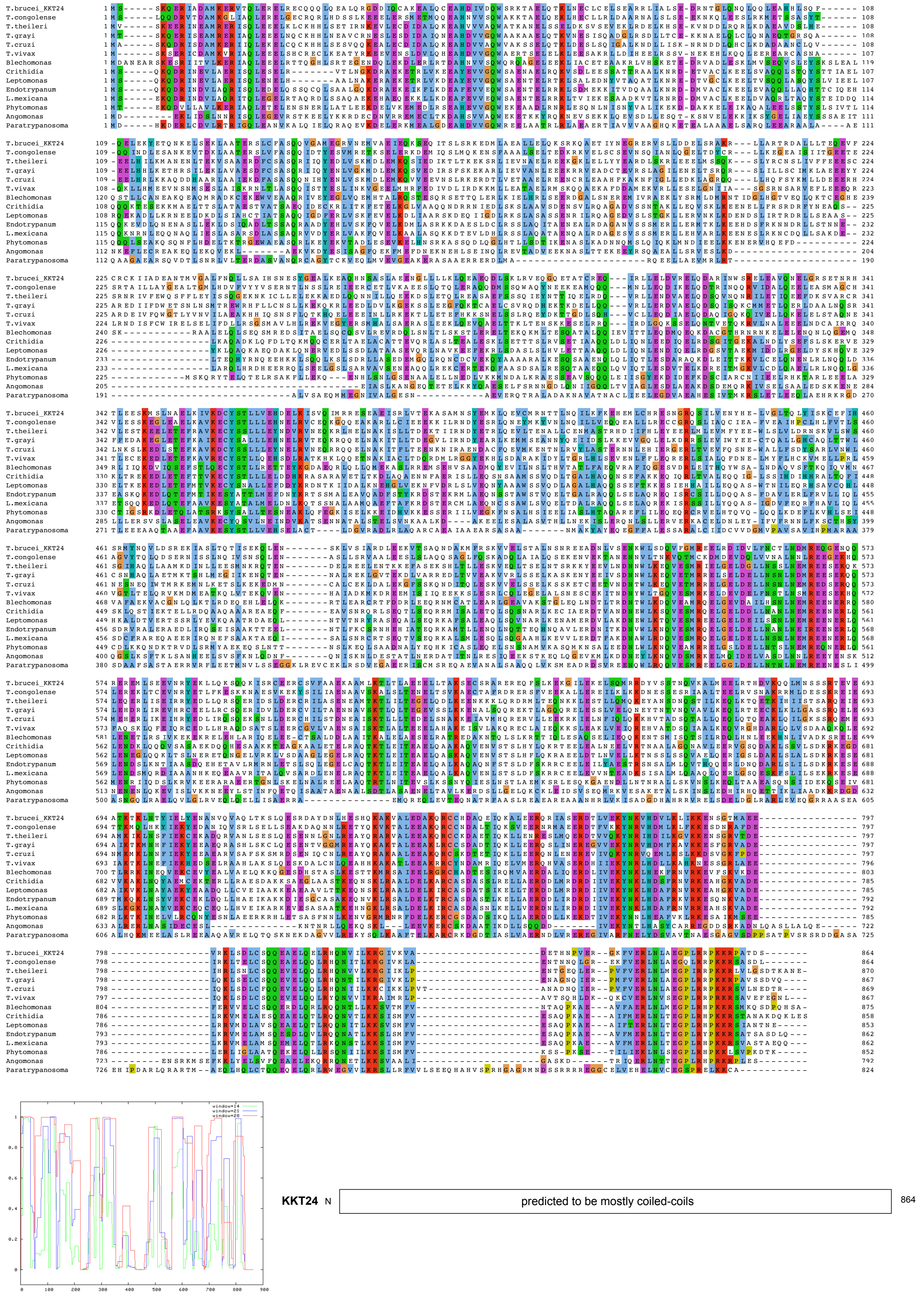

Figure S3. Multiple sequence alignment of KKT24 and its coiled-coil prediction

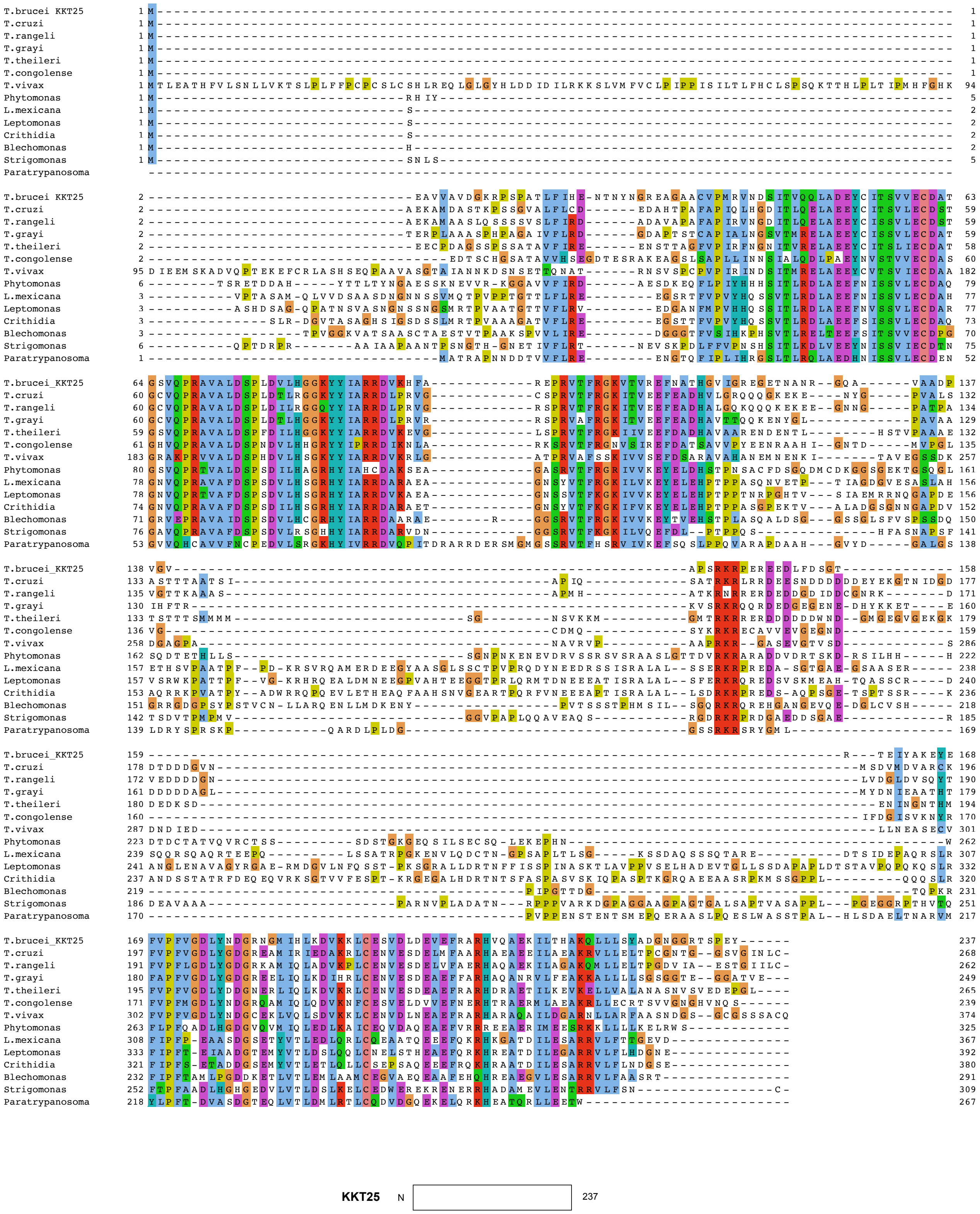

Figure S4. Multiple sequence alignment of KKT25

Supplemental Tables

Table S1. Lists of all proteins identified in the immunoprecipitates of KKT22–25, KKT2, and KKIP1–7 by mass spectrometry (Excel file).

Proteins identified with at least two peptides are shown. Common contaminants are marked by asterisk. Lack of KKIP2–7 peptides in the immunoprecipitate of KKIP1 as well as lack of KKIP1 peptides in the immunoprecipitates of KKIP2–7 are highlighted in green.

Table S2. Trypanosome cell lines used in this study.

Name	Description
SmOxP9	Parental cell line that expresses TetR and T7 RNAP (Poon et al., 2012)
BAP412	heterozygous Δkkt3 (this study)
BAP1123	KKT3-YFP/Δkkt3 (this study)
BAP567	tdTomato-KKT2 (neomycin) (this study)
BAP1490	YFP-KKT22 (this study)
BAP1454	YFP-KKT22, tdTomato-KKT2 (neomycin) (this study)
BAP1549	YFP-KKT23 (this study)
BAP1593	YFP-KKT23, tdTomato-KKT2 (blasticidin) (this study)
BAP1635	YFP-KKT24 (this study)
BAP1819	YFP-KKT24, tdTomato-KKT2 (blasticidin) (this study)
BAP1742	YFP-KKT25 (this study)
BAP1820	YFP-KKT25, tdTomato-KKT2 (blasticidin) (this study)
BAP710	YFP-KKIP1 (Llauró et al., 2018)
BAP825	YFP-KKIP2 (this study)
BAP826	YFP-KKIP3 (this study)
BAP808	YFP-KKIP4 (this study)
BAP827	YFP-KKIP5 (this study)
BAP828	YFP-KKIP6 (this study)
BAP829	YFP-KKIP7 (this study)

Table S3. Plasmids used in this study.

Name	Description
pEnT5-Y	TY-YFP tagging vector, Hygromycin (Kelly et al., 2007)
pEnT6-tdTomato	TY-tdTomato tagging vector, Blasticidin (Kelly et al., 2007)

pPOTv7<br>-eYFP	Vector for PCR only tagging (POT) of target genes, Blasticidin (Dean et al., 2015)
pBA183	Neomycin gene cassette used for gene disruption (p2705 derivative) (Kelly et al., 2007)
pBA67	TY-tdTomato-KKT2 tagging construct, Hygromycin (Akiyoshi and Gull, 2014)
pBA164	TY-tdTomato-KKT2 tagging construct, Blasticidin (Nerusheva and Akiyoshi, 2016)
pBA809	TY-tdTomato-KKT2 tagging construct, Neomycin (this study)
pBA1715	TY-YFP-KKT23 tagging construct, Hygromycin (this study)
pBA1803	TY-YFP-KKT24 tagging construct, Hygromycin (this study)
pBA2004	TY-YFP-KKT25 tagging construct, Hygromycin (this study)
pBA928	TY-YFP-KKIP1 tagging construct, Hygromycin (Llauró et al., 2018)
pBA1226	TY-YFP-KKIP2 tagging construct, Hygromycin (this study)
pBA1227	TY-YFP-KKIP3 tagging construct, Hygromycin (this study)
pBA1236	TY-YFP-KKIP4 tagging construct, Hygromycin (this study)
pBA1228	TY-YFP-KKIP5 tagging construct, Hygromycin (this study)
pBA1229	TY-YFP-KKIP6 tagging construct, Hygromycin (this study)
pBA1230	TY-YFP-KKIP7 tagging construct, Hygromycin (this study)

Table S4. Primers and synthetic DNA used in this study.

To make	Primer and synthetic DNA sequence (all are listed in the 5'-to-3' direction)
BAP412	SmOxP9 was transfected with a fusion PCR product consisting of:<br>1. Upstream targeting sequence proximal to KKT3, amplified from genomic DNA using primers BA909 and BA911,<br>2. Neomycin gene cassette amplified from pBA183 using primers BA903 and BA904, and<br>3. Downstream targeting sequence distal to KKT3, amplified from genomic DNA using primers BA914 and BA915. Clones were screened by PCR as follows<br>BA941 and BA943: 1.4 kb in strains if carrying the deletion<br>BA942 and BA943: 0.9 kb in strains if carrying the deletion<br>BA909: GTGATGGTGTTGCATATATATAT<br>BA911: TGGGAACTTAGTCAACCTCCTCGACTTTAGGGCGCTTTACTGGTAATATATAA<br>BA903: CCTAAAGTCGAGGAGGTTGA<br>BA904: CTCGATAAATAAATAGAAGTGC<br>BA914: CAACAAAGCACTTCTATTTATTTATCGAGCATGCCTGTTTGTGCAGCTT<br>BA915: GTACCCAAAGTGGAAAAAAG<br><br>BA941: TGCTGAAGCATCCGCTGATA<br>BA942: CCACCTATCTGCTGAAGTTG<br>BA943: GTCGGTCTTGACAAAAAGAA
BAP1123	PCR-based C-terminal YFP tagging of KKT3 using pPOTv7 (eYFP, blasticidin) on BAP412<br>BA1821:<br>GTAATGGAGTTTGTGAGGTGCTTGATGAGGAAAAATTCCCCCTTTCGGAGGAACTCAACCAGATGCTCTACGGTG<br>GCGTGGGTTCTGGTAGTGGTTCC<br>BA1822:<br>GAAATGCGACAGCAGACGGAAACGGAAAAAAAAATAAAAAAAAAGAGAGGGCTATCTGTAATTCTTTACGTAC<br>ATCACTTCCAATTTGAGAGACCTGTGC
BAP1454<br>and	PCR-based N-terminal YFP tagging of KKT22 using pPOTv7 (eYFP, blasticidin) on BAP567 (for BAP1454) or SmOxP9 (for BAP1490)<br>BA2129:<br>TTGCCAATTCCACATTCGTGCATTTGGTGTTCCTCCTTTTTAACTAGACACATCACACCCCAGGCAACAGCCTAAA<br>AAGAGTATAATGCAGACCTGCTGC

BAP1490 BA2130:
GCGGCCTCCAAATTTTCCCGTGTACTATAAGGGGACATATCTCGCCTTTCGTGCCGTGAGGAAAAGCTGGAGCCT
ACCATACTACCCGATCCTGATCC

pBA1715 Synthetic DNA for the N-terminal tagging target sequence for KKT23 with XbaI and BamHI, cloned into pEnT5-Y

GATCGATCGATCTCTAGAGGAGCAGGTTTACTTAGTGATGAGCACCTGGCATTGCTGGCGAAGTACTACGCCAC
GGTAGAGTTCACAGGTGAACAGAAGGACGCGCTCATAGAAAAATACTGGGAGGCAAATGAAGCTGAGCGCAA
GGCCATCGCGAGGGCCTACGCATCGCTCTTTGCCAATGACGCCGACTTCATTCAGCGACTGCTTGCCCACTACG
ATATGCATGTTAGCCCGCATGTGAGTCAGGGTGCGAGCAGTTGCAATGAGAACGGCGGCCGCATCGACCACCC
ATCAATGCTTTGTACAAGAAGAGCATCTTTCCCCTTTCTTTTTTGTCCGACTCGGAAGGTGAAAGAAGATTGAAA
AGAGACAATATTGACAGCCATAGAGGGCTTGCAGCGTGGGATAGTGTGCGCACTGGGGGTGTACAAGTGGGTA
CGCAAAAAAATATTTCCCCCTCCCTCGAAATTTTGTTTCTGGCGTTTTCGTCACGCCTTTTTTTTTTTGTATTTGAG
GGTGCTGTGCATACGGATCCGATCGATCGATC

pBA1803 Synthetic DNA for the N-terminal tagging target sequence for KKT24 with XbaI and BamHI, cloned into pEnT5-Y

GATCGATCGATCTCTAGAGGAGCAGGTTCATCCAAGCAGGAGAGAATAGCCGATGCTATGAAGGAGCGCGTCAC
CCAACTAGAAAGGGAATTGAGGGAATGCCAGCAGCAGCTACAGGAGGCGTTGCAAAGAGGTGATGATATACAG
TGCGCCAAGGAAGCGTTACAGTGCGAAGCCCATGATATTGTGGATCAGTGGTCTAGGCGGCCGCCCGAATTGTTG
TTTTTCATCACTCACCTAATGTGGCATGGGAGTAATTCACATGATGTGACCCTACAAGGCGATCAAGATCTTTAC
AATAATAGCAATAATAATAATAGATTGTATGTGCCGTTATACTGTCATCCCTATCTCATTATCAACCGGTCTTGTG
TTGATGTATGGTACAACGAACCTCGGAAGTTTCCCTATAGTTTAACTACCACTTGCGATTGATATCCTCATCTTAT
TCTGCTTTAGTCGGATCCGATCGATCGATC

pBA2004 Synthetic DNA for the N-terminal tagging target sequence for KKT25 with XbaI and BamHI, cloned into pEnT5-Y

GATCGATCGATCTCTAGAGGAGCAGGTGAGGCTGTGGTCGCAGTGGATGGCAAACGTCCCAGCCCGGCGACTTT
GTTCATTCATGAAAACACTAACTATAATGGAAGGGAAGCGGGTGCGGCGTGCGTTCCCATGCGAGTCAACGATA
GCATTACTGTGCAACAACTCGCTGATGAGTACTGCATCACGAGTGTTGTGGAATGTGATGCCACGGGTAGTGTTC
AACCCCGAGCGGTGGCGCTAGATTCACCCCTTGATGTTCTCCACGGGGGAAAGTATTATATTGCCAGGCGTGATG
TGAAACATTTTGCGCGGGAACCCCGGGTAACATTCCGTGGAAAAGTTACCGTGAGGGAGTTTAATGCGACACAC
GGGGTAATTGGGCGGGAAGGGGAAACAAACGCGAACCGAGGACAAGCTGTCGCTGCGGATCCCGTAGGCGTAG
CCCCATCCCGCAAGCGGCCCGAACGGGAGGAAGACTTGTTCGATAGCGGCACACGCACTGAGATATATGCGAAG
GAGTACGAATTTGTGCCCTTCGTGGGGGATCTCTACAACGATGGAAGGAATGGCATGATTCATCTCAAAGATGTG
AAGAAACTCTGTGAAAGCGTAGATTTAGACGAAGTTGAGTTCAGAGCGCGACACGTACAAGCGGAGAAAATCCT
AACCCATGCAAAACAACTATTGCTTAGCTACGCGGACGGCAACGGCGGGAGGACCAGCCCCGAATACGCGGCCG
CGGCGTGGAGTGAAGTGCGTCATTGTGTGGTTGATCTTTGAGACGGAGTGTTACAGTTTCCTTTAAGGGGGGAAA
TGCTTGGTCTGCTTATTTATTATATCCACTCGTTTTAACGCTCCCGATAACCGCCAATGCGCCGCTTGGCGGGCAC
ACCGCCGCACACAAACAGGTGAGAAGACTCCCATATCATTTCTGATTCTCTTCTAGCTTTGGACTCAGTTACCTTA
AGGGAAGGAAGGGCAAGGGAACGTGGATCCGATCGATCGATC

pBA1226 Synthetic DNA for the N-terminal tagging target sequence for KKIP2 with XbaI and BamHI, cloned into pEnT5-Y

GATCGATCGATCTCTAGAGGAGCAGGTCCGAATTCGGCACCGATGAAAACAATACCGCCGAAGTCGCGTGTTCC
CCCCGATTGGATTCACCCGGCGCTGCATCGCCAGTGGCAGAGACGTGATAAATTGAGGACTCCACACGAATTGC
GACTTGAGGAATTAGATGTTCAACGAACGGAGATGGAGGAAGCGAGCCGCCGCATTATGAGCATTGTCTCAGAG
AAGAAGAGCGCACTTGAGAAGTTGGATCGTCAACGAGAGAAGGCAAAGGCAGGCGGCCGCATATATGTATATCT
ATATTTGTGTATACGTTGAGGGTGAACTCATCAATGTGTTACAATTTTTTCTTTTCTATTGCCCTCTTTTTTCTTTAT
TTGTGCGTTTCCACCCTACTAGTCGGTTCAATCCGAACCCGCACGCCTCTTTTTCCCCTTCTATCATTTATCATTAC
ATCACTGCTTCGCACTTTTATAACCGTCGTTGTTGGTTTGCTTATTTGCTGCTTGTGTCGCTGATCCGTTACGTGCT
TGAGGGATCCGATCGATCGATC

pBA1227 Synthetic DNA for the N-terminal tagging target sequence for KKIP3 with XbaI and BamHI, cloned into pEnT5-Y

GATCGATCGATCTCTAGAGGAGCAGGTGCTGGTGCGGAACAACGACAATTTCACAGCGTAGAGGAAGTTAGCGT
AGAGTTAGGTCGCGCATCACTGCATGCTTCTTGGGGTTTCAAGACATATGACGGTGTCTGCCCCCTTCGTGTACGT
GATGTAGCAGCGGAGATTCCTGAAGGTGTAAGGCGCGGTGATGAAATAATTGTAATTAACGGAATCCGACCAGG
GAGCTATGATGAGGGAATGTCATTGCTGCATCAGGCGCAGTCAACCGTTCGCGGCCGCACGTGGAGCCCGTACG
CCAAGGAGGTAAACCAAAAAGAGGGGGAAAATAACGAAGAGCAGGGACAGCAAGAACACGTTTTGGGATTTCG
AGGAATAAGTTCAATTTGGTGTCGGCTACGTTTGATCGTTTACGAAGGGTAGAAAAAAGTTCGTAGTAACGACCG
TACAACAAAAGGTAGCTGGGAAAAAATTGATTGAGTGACACGATTTTGTCATTGCGGCGGAAAACATTAATTCG
ATCGGCCACGAGGGATCCGATCGATCGATC

pBA1236 Following two PCR fragments were cloned into pEnT5-Y using XbaI and BamHI

- KKIP4 CDS targeting sequence with XbaI and NotI
BA1700: GATCGATCTCTAGAGGAGCAGGTTGGAACGCATTTAGCGG
BA1701: GATCGATCGCGGCCGCGCTCATCGATATTAGGCTGG
- KKIP4 5'UTR targeting sequence with NotI and BamHI
BA1702: GATCGATCGCGGCCGCATGAAGTGTCTCCTTGCTAC
BA1703: GATCGATCGGATCCCTTATCGCAGCGAAGAAAAG

pBA1228 Synthetic DNA for the N-terminal tagging target sequence for KKIP5 with XbaI and BamHI, cloned into pEnT5-Y

GATCGATCGATCTCTAGAGGAGCAGGTGACAGTGATACCATTTATTGTGATGAAAGCTCCGTAGCGAGTCTTTCA
CAGCCTCAAGGCCGTCCCGCGTTACGACTCGATTCCCTCCCCCCTACACCAATGGTCCTAACGCCATGTGACTCC
AACGTCACGGCATCCGCCAGGAAAAATCAACTAAAGCAGCAACGGCACCAGCACCGGTCATCATTTGCATGCAC
GGCGCTAACATCACCTTCACTTTCGCAGTCCGACTTCATACCTGAGACTCGCGGCCGCGGCACACCCGCGCATAC
ATCCCTCGGTTCTATACTCCTACTTGTGCATCGTCAAGTGAAGGAGGCACTTGTTTATAACATATATATATATATA
TATATATTATACGTTGCCGCCTCACTTCCATTTTGCGGTCATCGTCATCATTCATTCGCTTGAGCGAGCGGTTGTG
CTCACACAATAAAAATCAACAACAGTGTTGTACTGAGGAGAAACGAAGGGAAGGGGCATCGGTGGCAACAGCG
CATTGACAGGATCCGATCGATCGATC

pBA1229 Synthetic DNA for the N-terminal tagging target sequence for KKIP6 with XbaI and BamHI, cloned into pEnT5-Y

GATCGATCGATCTCTAGAGGAGCAGGTTCAACAGAGGAACTCGTGCAGCGGGTTGTACAAATGCAAATGACGAG
TCCTCATTTTGCGGAATATTGCGTTGCTCACGTCACAGATTCCAAACATAAGTTTTTATTCGATGCAACAGATAGT
GAAGAGAGGCGACTTTACTGCGCGCTTCTTCAGAGGATGAGGGGGGGATTTATTAGTGACGAGTCGAGGGAATG
TGCGGGAGCGACGGCGGCAGGAACGGAGCGTCAGCGGCAGCGGCATCGGCGCGGCCGCGCCAACATTTAAGGT
GAAGATTTTTGTTGTTTTTTTTTTGATTTGTTTACATTCAAATTATAATTAGTTTCACATTTATTCATTTAAAGTTGT
TGAATTAAATCGGGTGCGATAGGGTATTACACACGGGAGCAAACGCATTACGGTGAATTGCTTAACGTGTTTGAA
ACTTTTGCTCTGACGAGAGTTCGGATAAGAAGTTGTGTTGTGTGGATTAGGACGGACAGACAAGAAAAAAAAAA
AGAAAATTGGATCCGATCGATCGATC

pBA1230 Synthetic DNA for the N-terminal tagging target sequence for KKIP7 with XbaI and BamHI, cloned into pEnT5-Y

GATCGATCGATCTCTAGAGGAGCAGGTTCATCGTCAGAAGCGGTGAAGGCGCTTGCATCCCTGACGAAGGAAGA
GCTCATGCAGCGCGTGTTAGAGCTGCAAGGGAAGAATGCGGAGTTGTACGACGAGGTAGAACAGTTGCGGCAGC
GCCTCTCGCAAAACAGGATTCCGGACGTCAGCAACCCTCGTGTTTCGATTCGCCATTCGTGTGATGTCGGTTCGTG
TTCGCCCAGCAGGTTCAGTACGCTGGCAAGTAGCGTGGGGGGACAATACGGCGGCCGCGTCTTCTTTCGCCTGGT
GTGTGCTTTTTATTCTCTACCCCATTATTCTTCGCAAGCACTCTAGTTAACTATTTTTTATTTCTTTTTTTTACGTCT
CTGGGGACGGCAGGGACGTTTTGTGCGTTGCTGTCGTATTGAGTCTTAACACAGAGACCAAGAAAGAGGGACAA
TAGCCGCAATCTATACAGTATTTGTTGCCCAGATACGAGCGAGGGCCGGGAGAATATACATACTGGCATATAGG
CGGAGCGGGATCCGATCGATCGATC

Supplemental References

Akiyoshi, B., and K. Gull. 2014. Discovery of unconventional kinetochores in kinetoplastids. *Cell*. 156:1247–1258. doi:10.1016/j.cell.2014.01.049.

Dean, S., J. Sunter, R.J. Wheeler, I. Hodkinson, E. Gluenz, and K. Gull. 2015. A toolkit enabling efficient, scalable and reproducible gene tagging in trypanosomatids. *Open Biol*. 5:140197. doi:10.1098/rsob.140197.

Kelly, S., J. Reed, S. Kramer, L. Ellis, H. Webb, J. Sunter, J. Salje, N. Marinsek, K. Gull, B. Wickstead, and M. Carrington. 2007. Functional genomics in *Trypanosoma brucei*: a collection of vectors for the expression of tagged proteins from endogenous and ectopic gene loci. *Mol. Biochem. Parasitol.* 154:103–109. doi:10.1016/j.molbiopara.2007.03.012.

Llauró, A., H. Hayashi, M.E. Bailey, A. Wilson, P. Ludzia, C.L. Asbury, and B. Akiyoshi. 2018. The kinetoplastid kinetochore protein KKT4 is an unconventional microtubule tip-coupling protein. *J. Cell Biol.* 217:3886–3900. doi:10.1083/jcb.201711181.

Nerusheva, O.O., and B. Akiyoshi. 2016. Divergent polo box domains underpin the unique kinetoplastid kinetochore. *Open Biol*. 6:150206. doi:10.1098/rsob.150206.

Poon, S.K., L. Peacock, W. Gibson, K. Gull, and S. Kelly. 2012. A modular and optimized single marker system for generating *Trypanosoma brucei* cell lines expressing T7 RNA polymerase and the tetracycline repressor. *Open Biol*. 2:110037. doi:10.1098/rsob.110037.
