# Supplementary material for: Identification of four unconventional kinetoplastid kinetochore proteins KKT22–25 in Trypanosoma brucei
Source: Open Biol. 2019 Dec 4;9(12):190236. doi: 10.1098/rsob.190236 (PMC6936259; doi:10.1098/rsob.190236)
Supplement: Reporting form [file rsob190236supp3.pdf]

OPEN BIOLOGY

Completed by:

Bungo Akiyoshi

Open Biology reproducibility reporting form

Authors are expected to complete this form to ensure good reporting standards and reproducibility in your paper. The form is intended for publication alongside accepted manuscripts. The table will serve as an overview of the key reagents, cell lines, source data and software used in the study should provide all the experimental and statistical details to allow others to replicate their study. The form is based on the NIH Principles and Guidelines for Reporting Preclinical Research and is intended to help ensure high standards for reporting and to aid reproducibility. Items in the table must also be reported in the 'methods' section of the manuscript within the context of their use. Any references cited in the reporting form must be included in the References list of the manuscript.

If you have any questions, please consult our author information or publishing polices page - or contact us: openbiology@royalsociety.org.

Definitions:

**Resource or reagent type -** Provide full descriptive names of items so can be identified (include version number for software, host source for antibody, strain name etc)	**Source** – *report the stockist, manufacturer, company, lab (name of principle investigator)*	**Identifier** - *include the catalogue number, accession or database number*

Open Biology recommends RRIDs as the identifier (in particular for antibodies and organisms, but also for software tools and databases). For more details on how to obtain or generate an RRID for existing or newly generated resources, please search for RRIDs.

Please indicate if the following were used in the study:

Cell lines

- [x] Yes (please provide further details in table below)
- [ ] Not applicable

Resource or reagent type	Source	Identifier
Trypanosoma brucei brucei, procyclic form	Keith Gull lab	SmOxP9

Antibodies

For antibodies, provide a citation, catalogue number and/or clone number and batch number. We recommend using RRIDs as the identifier; more details on how to obtain or generate an RRID for existing or newly generated resources, please search for RRIDs.

- [x] Yes (please provide further details in table below)
- [ ] Not applicable

Resource or reagent type	Source	Identifier
monoclonal anti-GFP antibodies	Roche	11814460001

Plasmids

☑ Yes (please provide further details in table below)

☐ Not applicable

Resource or reagent type	Source	Identifier
All details are listed in Table S3		

Bacterial or viral strains

☐ Yes (please provide further details in table below)

☑ Not applicable

Resource or reagent type	Source	Identifier

Recombinant proteins

☐ Yes (please provide further details in table below)

☑ Not applicable

Resource or reagent type	Source	Identifier

Primers

☑ Yes (please provide further details in table below)

☐ Not applicable

Resource or reagent type	Source	Identifier
All details are listed in Table S4		

Recombinant DNA

☐ Yes (please provide further details in table below)

☑ Not applicable

Resource or reagent type	Source	Identifier

Commercial assays

☐ Yes (please provide further details in table below)

☑ Not applicable

Resource or reagent type	Source	Identifier

Biological samples

☐ Yes (please provide further details in table below)

☑ Not applicable

Resource or reagent type	Source	Identifier

Animal participants

Please report species, strain, age and sex for laboratory animals (or age, sex and species for field animals). See our guidelines on research ethics - follow ARRIVE guidelines

☑ Not applicable

Human participants

Please provide relevant data on human research participants including age, gender, genotypic information. See our guidelines on research ethics - follow ARRIVE guidelines

☑ Not applicable

Software

Describe the software used to analyse the data in this study. For manuscripts using custom algorithms or software not yet described in the published literature, software must be made available to editorial office and reviewers upon request.

☑ Not applicable

Availability of Materials

Indicate whether there are restrictions on availability of unique materials or if these materials are only available for distribution by a third party.

- [x] No restrictions
- [ ] Restrictions apply (please provide further details below):

Sample size

Describe the statistical methods that were used to predetermine sample size or how sample sizes were chosen

Statistical analyses and parameters

All figures and tables that use statistical methods, please confirm that the following items are present in relevant figure legends:

- exact sample size (n) for each experimental group or condition, given as a number, not a range.
- A description of the sample collection that allows the reader to understand whether the samples represent technical or biological replicates (including how many animals, cultures, etc.).
- A statement of how many times the experiment shown was replicated in the laboratory.
- A clear description of statistics including central tendency (e.g. median, mean) and variation (e.g. standard deviation, interquartile range) - Statistical test results, e.g. P values.
- Details of statistical method.

- [ ] I/we confirm
- [x] Not applicable

Additional relevant information not included in the above:
